# The Reactome pathway knowledgebase

**DOI:** 10.1093/nar/gkt1102

**Published:** 2013-11-15

**Authors:** David Croft, Antonio Fabregat Mundo, Robin Haw, Marija Milacic, Joel Weiser, Guanming Wu, Michael Caudy, Phani Garapati, Marc Gillespie, Maulik R. Kamdar, Bijay Jassal, Steven Jupe, Lisa Matthews, Bruce May, Stanislav Palatnik, Karen Rothfels, Veronica Shamovsky, Heeyeon Song, Mark Williams, Ewan Birney, Henning Hermjakob, Lincoln Stein, Peter D'Eustachio

**Affiliations:** ^1^European Bioinformatics Institute (EMBL-EBI), European Molecular Biology Laboratory, Wellcome Trust Genome Campus, Hinxton, Cambridge CB10 1SD, UK, ^2^Ontario Institute for Cancer Research, Toronto, ON M5G0A3, Canada, ^3^College of Pharmacy and Health Sciences, St. John’s University, Queens, NY 11439, USA, ^4^NYU School of Medicine, New York, NY 10016, USA, ^5^Cold Spring Harbor Laboratory, Cold Spring Harbor, NY 11724, USA and ^6^Department of Molecular Genetics, University of Toronto, Toronto, ON M5S 1A1, Canada

## Abstract

Reactome (http://www.reactome.org) is a manually curated open-source open-data resource of human pathways and reactions. The current version 46 describes 7088 human proteins (34% of the predicted human proteome), participating in 6744 reactions based on data extracted from 15 107 research publications with PubMed links. The Reactome Web site and analysis tool set have been completely redesigned to increase speed, flexibility and user friendliness. The data model has been extended to support annotation of disease processes due to infectious agents and to mutation.

## INTRODUCTION

At the cellular level, life is a network of molecular reactions that can be organized into higher order interconnected pathways. Molecules are synthesized, degraded, transported from one location to another and assembled into complexes and higher order structures with other molecules. Intensive studies of cellular signaling, motility, vesicular trafficking and other aspects of cell biology, coupled with the development of comprehensive catalogs of human genes and their protein products, have enabled the description of many cellular processes in the same molecular detail that has been a standard for metabolic processes for a generation. By annotating all of these processes in a single, consistent reaction-pathway format, the Reactome Knowledgebase systematically links human proteins to their molecular functions, providing a resource that functions both as an archive of biological processes and as a tool for discovering unexpected functional relationships in data from gene expression pattern surveys or somatic mutation catalogues from tumor cells (e.g. 1–3).

Since its inception 10 years ago, Reactome has grown to include (version 46–September 2013) annotations for 7088 of the 20 774 protein-coding genes in the current Ensembl human genome assembly (34% coverage), 15 107 literature references and 1421 small molecules organized into 6744 reactions collected in 1481pathways. Notable recent additions include extensive annotations of phospholipid and eicosanoid metabolism, protein glycosylation and SCF-KIT, IGF1R, NOTCH and HIPPO signaling, as well as annotations of regulatory processes mediated by non-coding RNAs.

Here, we will focus on three new features of Reactome: the development of a strategy to annotate the disease counterparts of normal human processes, the deployment of a redesigned Web site and the extension of tools for data analysis.

### Disease curation using an enhanced data model

The Reactome data model (4) builds on earlier work by Kanehisa *et al.* (5) and Karp *et al.* (6) to classify and catalog physical entities (proteins and other macromolecules, small molecules, complexes of these entities and post-translationally modified forms of them), their subcellular locations and the transformations they can undergo (biochemical reaction, association to form a complex and translocation from one cellular compartment to another). The central class of the Reactome data model is a *Reaction*, and subclasses of *Reaction* model these core biological events. Reactions are grouped into pathways, which in turn are assembled into a hierarchy of biological processes. Wherever appropriate, Reactome entities are linked to external reference databases such as UniProt (7), Ensembl (8), ChEBI (9) and Rhea (10). The full Reactome database schema is available at http://www.reactome.org/cgi-bin/classbrowser?DB=gk_current.

A straightforward definition of disease allows us to annotate a broad range of major disease processes at the molecular level. Diseases that can now be annotated in Reactome arise in one of three ways: a mutation, somatic or germ-line, leads to a non-functional gene product so processes that normally depend on that gene product do not take place; a mutation leads to a gene product with a novel function, enabling novel reactions whose products perturb normal human processes; or an infectious agent such as a virus introduces novel gene products whose novel reactions perturb normal human processes.

To identify disease-associated entities and events, a new ‘disease’ attribute is added, taking its value terms from a disease ontology [currently http://disease-ontology.org/(11)]. This attribute is multivalued, so an entity or event that has roles in multiple disease processes can be annotated to capture all of those roles.

With this addition, diseases due to infection can be annotated within our data structure. Our existing ‘species’ attribute allows pathogen-derived proteins, DNA and RNA to be distinguished from molecules encoded in the human genome. The ‘species’ attribute, also associated with complexes, reactions and pathways, can be multivalued, allowing complexes containing both host and viral proteins or reactions involving host and viral components to be properly identified. Finally, addition of Gene Ontology (GO) (12) host_cell terms to our cell compartment vocabulary allows us to localize pathogen-derived entities accurately, in compliance with GO annotation practice.

To denote molecular properties of a protein modified by a somatic or germline mutation in the gene that encodes it, as opposed to post translational modification, we created a new class. This class of genetic modifications has subclasses to accommodate substitution of a canonical residue by a different one, the insertion or deletion of multiple contiguous residues into a canonical sequence and the generation of a fusion protein containing fragments of two canonical ones (13). The underlying chemical similarity between a protein that differs from its canonical form due to a mutation and one that differs due to co- or post-translational modification allows us to maintain compliance with the PSI-MOD standard for annotation of protein modifications (14).

Reactions involving mutated proteins as catalysts, inputs, outputs and regulators are annotated exactly as wild-type reactions are. To link reactions involving a mutated protein to those involving its normal counterpart, an optional ‘normal reaction’ attribute is used. A reaction in which a receptor constitutively activated by a mutation transmits a signal is thereby paired with the wild-type reaction in which a normal receptor is activated by ligand binding. A reaction catalyzed by the normal form of an enzyme is paired with the different one catalyzed by its gain-of-function mutant counterpart or with a dead-end reaction (normal inputs, no outputs) associated with its loss-of-function mutant counterpart. A limitation of this strategy at present is that, as Reactome does not capture quantitative data such as reaction rates or binding affinities, quantitative effects of mutations are not readily annotated.

**Figure 1.**
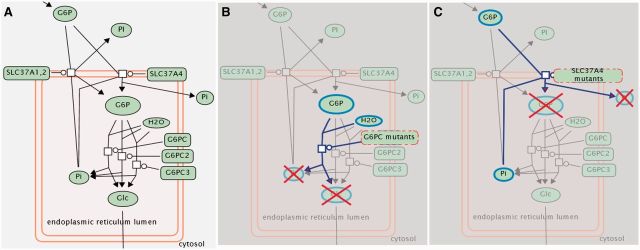
Example of disease curation and visualization in Reactome. The normal process of glucose export from the liver under fasting conditions (**A**) is disrupted by mutations that block glucose-6-phosphate hydrolysis within the endoplasmic reticulum (**B**) or the transport of glucose 6-phosphate and orthophosphate (Pi) between the endoplasmic reticulum and the cytosol (**C**).

At the level of our event hierarchy, these normal-disease pairings support a disease event hierarchy that parallels our normal event hierarchy, a useful and generalizable organization. These pairings also support a visualization scheme that highlights the relationship between the normal and disease processes. As described previously (15), the physical entities and their interactions that comprise a pathway are laid out in a pathway diagram that follows the SBGN process description language (http://www.sbgn.org/Documents/Specifications) and that is displayed on our Web site. The software that generates these displays has been extended to create disease displays in which the variant forms of the events responsible for a disease process are superimposed and highlighted on the normal process diagram (Figure 1).

We have used these extensions of the Reactome data model and pathway visualization process to create a new disease pathway classification in our event hierarchy that incorporates existing material such as the HIV and influenza life cycles, amyloid formation and botulinus toxin neurotoxicity, together with new material that includes malignant transformation due to mutations in the EGFR and FGFR signaling pathways and mucopolysaccharidoses. Our current release includes annotations for 420 mutant forms of 68 proteins.

### Updated Reactome Web site

Our home page has been redesigned completely to support intuitive access to our pathway browsing and data analysis tools. The new Web site retains a top menu bar to provide easy access to all of our tools and resources, accompanied by a central panel of links to our most widely used tools and a footer that displays all tools and resources. This organization is consistent with the general model for resources associated with EBI. News is now available as an interactive Twitter display, which also provides open real-time feedback, both by the Reactome group and our user community.

### Dynamic pathway portal

**Figure 2.**
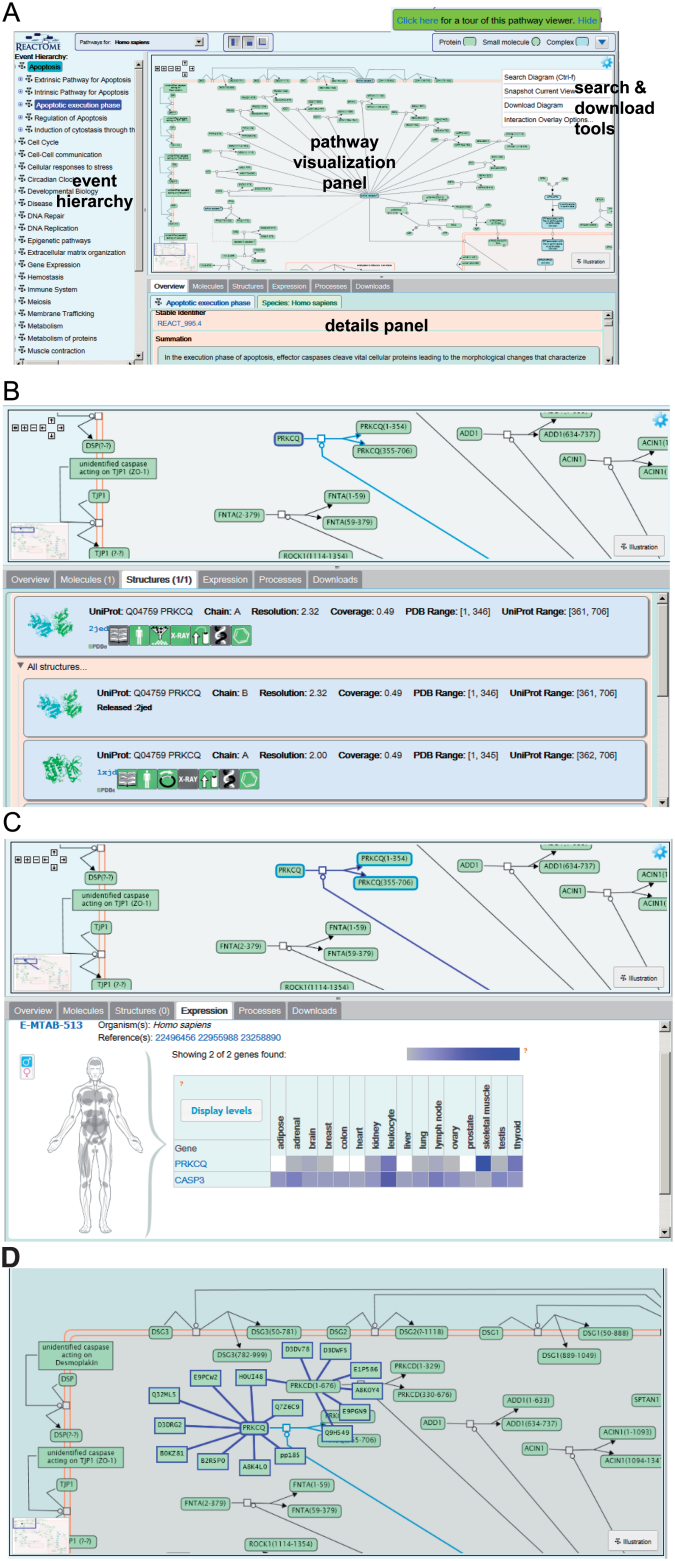
Visualization of the execution phase of apoptosis. (**A**) Choosing the entry for this event from the event hierarchy in the left panel of the web page causes the pathway to be displayed in a large panel to the right, laid out in SBGN process description format. Buttons at the top of the panel open a diagram key and give access to a brief tour of features of the web page. The panel at the bottom of the page contains text descriptions of various features of the pathway and participating molecules, linked to external databases. (**B**) The user has selected the reaction ‘Caspase 3-mediated cleavage of PKC delta’; the ‘Structures’ detail tab displays PDB 3D structural data and citations for proteins in that reaction and the ‘Expression’ detail tab (**C**) displays condition-specific gene expression data from the Gene Expression Atlas. (**D**) The molecular interaction (MI) overlay displays proteins that interact with user-specified proteins PRKCQ and PRKCD.

We have improved the flexibility and performance of our pathway browser by creating a new pathway diagram visualization tool using the canvas element introduced in HTML 5. The canvas element is used to render whole pathway diagrams in a Google map-like way with XML-encoded pathway diagram data retrieved from the server using a RESTful API (Figure 2A). The new diagram visualization tool offers quicker performance and better data overlaying technologies (see later in text) and bypasses the slow step to generate static images during database release.

The event hierarchy panel to the left has been redesigned to provide interactivity and access to the entire listing of all the Reactome pathways. Icons now indicate whether a pathway is new (N) or updated (U), and identify ones that are parts of disease processes (+). Navigation controls in the upper left corner enable zooming and panning across the pathway panel. A diagram thumbnail in the lower left shows the part of the pathway currently displayed in the visualization panel. A widget icon in the upper right corner of the panel links to tools for searching within the displayed pathway, overlaying the pathway with functional interactors (see later in text) and for downloading the diagram as a snapshot or PNG file.

A new tabbed ‘Details’ panel, below the pathway diagram, provides additional graphical and textual information. The ‘Overview’ tab provides summary information relating to the pathway, reaction or entity selected. The ‘Molecule’ tab displays external annotation and linking-out to other bioinformatic resources describing the selected molecule. The ‘Structures’ tab (Figure 2B) displays 3D structural data and citations for proteins from PDB (16), for small molecules from ChEBI and for the stoichiometry of metabolic reactions from Rhea. The ‘Expression’ tab (Figure 2C) serves condition-specific gene expression data from the Gene Expression Atlas (17). The ‘Processes’ tab displays Reactome pathways and reactions associated with the selected pathway node or event. Finally, the ‘Download’ tab allows users to download the description of the pathway in a variety of formats compatible with third-party tools.

Based on usability testing and feedback from our user community, we modified the context sensitive menus. In this new version, the mouse cursor can be positioned on the pathway background and right-clicked to display a new set of pathway diagram options.

The molecular interaction overlay that provides the display of proteins or small molecules that interact with Reactome pathway proteins (Figure 2D) is revised to include additional interaction databases through the PSIQUIC registry (18) including BindingDB (19), DrugBank (20) and GeneMANIA (21).

### Unified pathway analysis portal

**Figure 3.**
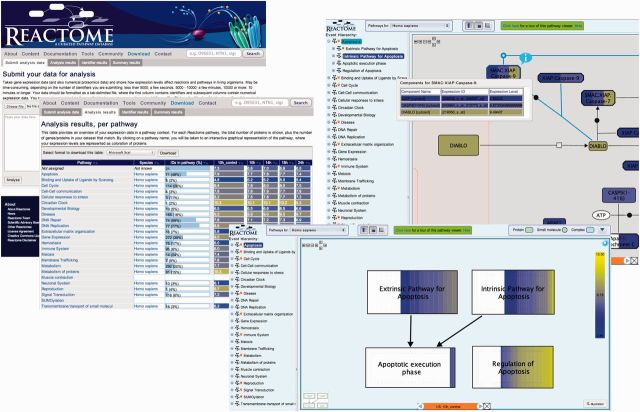
Pathway analysis. The data analysis workflow proceeds in three steps: data submission, a tabular display of results and visual display of results as an overlay in a pathway browser window. The fourth pane shows a detail of the pathway browser window, to highlight the display of expression levels.

We have merged pathway identifier mapping, over-representation and expression analysis tools into a single tabbed data analysis portal with integrated visualization and summary features (Figure 3). The pathway data analysis module accepts gene lists or expression data with numerical values (e.g. expression, abundance, fold expression change and quality scores). It will automatically distinguish between tab-delimited, comma-delimited or Microsoft Excel files and can cope with files that have been ZIP compressed.

The results of an analysis are displayed in three tabs that provide three different views of the same data. Pathway-oriented expression analysis provides a table of top-level pathways plus information relevant to the type of data submitted by the user. If presented with a list of identifiers, an enrichment analysis will be shown; numerical data will be summarized in a graphical way, using color to indicate average expression values. The identifier mapping view lists each of the identifiers supplied by the user with the corresponding UniProt identifier and a list of pathways in Reactome in which it functions. The overview is a completely new feature. It provides a canvas-based aggregated summary of the entire set of Reactome pathways, using visual cues to inform the user about the number of participating entities, relations between the pathways, average expression levels and other details.

The pathway names in the pathway-oriented view are clickable links, which take the user to the relevant pathway diagram. In it, the sub-pathway nodes, complexes and entities are colored according to expression level. Entities that lack gene expression information are colored white. Pathway and complex nodes are colored in vertical segments with each segment representing a protein in the pathway or a component of the complex. The segments are stacked in order from lowest to highest expression level. A new icon at the base of the diagram allows the user to single step through the individual experiments or time points.

### Community relationships and data exchange

Reactome continues to collaborate with other data resources, such as GO, NCBI, EBI and WikiPathways (22). Reactome provides a series of link-outs to many online bioinformatics resources from its protein pages; we have added links to GeneCards annotations (23). Reactome is open-source and open-data, and we have continuously supported the major open-data standards in the domain, including BioPAX levels 2 and 3 (24), PSI MITAB (18), Protégé (http://protege.stanford.edu), SBML-ML (25) and SBGN export format. Reactome now provides an SBGN file format generated using libSBGN for individual pathways. The Reactome SBML export has been upgraded to Level 2, Version 4 and is enriched with a wide variety of additional annotations, including Systems Biology Ontology terms (26). Reactome also supports the Protein Ontology in developing an ontology for protein modifications and protein complexes (27). Our new RESTful API provides outside users with direct access to pathway data in Reactome.

The Reactome data model has been adopted by the Gramene group for manual annotation of plant pathways, especially metabolic processes specific to plants. The current release of Plant Reactome (http://plants.reactome.org) includes 131 rice pathways.

Since 2011, Reactome has participated in the Google Summer of Code program as part of the Genome Informatics group, helping to create the software components for the new pathway browser, RESTful API and the pathway overview. The Reactome data and source code continues to be publically accessible under the terms of a Creative Commons Attribution 3.0 Unported License.
